# Cyclic AMP signaling in bone marrow stromal cells has reciprocal effects on the ability of mesenchymal stem cells to differentiate into mature osteoblasts versus mature adipocytes

**DOI:** 10.1007/s12020-012-9717-9

**Published:** 2012-06-14

**Authors:** Richard Kao, Weidar Lu, Alyssa Louie, Robert Nissenson

**Affiliations:** 1 University of California, San Francisco, San Francisco, CA USA; 2 Veterans Affairs Medical Center, San Francisco, CA USA

**Keywords:** Cyclic AMP (cAMP), Osteogenesis, Adipogenesis, Parathyroid hormone (PTH), Mesenchymal stem cells, Alkaline phosphatase

## Abstract

Stimulatory G protein-mediated cAMP signaling is intimately involved in skeletal homeostasis. However, limited information is available on the role of the cAMP signaling in regulating the differentiation of mesenchymal stem cells into mature osteoblasts and adipocytes. To investigate this, we treated primary mouse bone marrow stromal cells (BMSCs) with forskolin to stimulate cAMP signaling and determined the effect on osteoblast and adipocyte differentiation. Exposure of differentiating osteoblasts to forskolin markedly inhibited progression to the late stages of osteoblast differentiation, and this effect was replicated by continuous exposure to PTH. Strikingly, forskolin activation of cAMP signaling in BMSCs conditioned mesenchymal stem cells (MSCs) to undergo increased osteogenic differentiation and decreased adipogenic differentiation. PTH treatment of BMSCs also enhanced subsequent osteogenesis, but promoted an increased adipogenesis as well. Thus, activation of cAMP signaling alters the lineage commitment of MSCs, favoring osteogenesis at the expense of adipogenesis.

## Introduction

Fibrous dysplasia (FD, OMIM 174800) of bone is one of the manifestations of the McCune–Albright syndrome. Patients with McCune–Albright syndrome have activating missense mutations in GNAS, the gene encoding the α subunit of G_s_ [1–3]. These mutations have been demonstrated in various tissues, including bones [3–5] and samples from the monostotic form of fibrous dysplasia [6, 7]. In fibrous dysplasia, the expression of G_s_ protein and its transcript is upregulated during the maturation of precursor osteogenic cells to mature osteoblasts [8]. Examination of the fibrotic area revealed an excess of cells with features of pre-osteoblasts, and the abnormal cellular features of fibrous dysplasia could be reproduced in vitro by the addition of excess exogenous cAMP to human osteogenic cells or by stable expression of constitutively active form of G_s_α in human BMSCs [8, 9].

The role of the G_s_/cAMP pathway in bone formation is further highlighted by several findings. Conditional deletion of the α subunit of G_s_ in osteoblast lineage cells resulted in reduced trabecular bone formation [10]. Targeted expression in osteoblasts of constitutively active G_s_-coupled G protein-coupled receptors (GPCRs) resulted in markedly increased trabecular bone mass [11, 12], whereas expression of a constitutively-active G_i_-coupled receptor produced trabecular osteopenia [13]. Furthermore, expression of an engineered constitutively active G_s_-coupled receptor under the control of the osteoblast-specific Col1α1 2.3-kb promoter fragment in mice produced cellular features similar to that of the polyostotic fibrous dysplasia of McCune–Albright’s syndrome [11]. Alteration of G_s_ and G_i_ signallings in osteoblast lineage cells was able to generate striking, but opposite effects on skeletal tissues.

Several extracellular regulators of skeletal function are known to exert their actions through GPCRs, which are located in the osteoblast membrane. The best-studied GPCR that regulates skeletal function is parathyroid hormone 1 receptor or PTH1R [14]. This receptor is coupled to multiple G proteins, including G_s_, G_q_, and possibly G_i_, resulting in the activation of diverse downstream effectors [15–18]. Although PTH activates multiple signaling pathways, several lines of evidence indicate that PTH peptide fragments that specifically stimulate G_s_ signaling coupled to PTH1R could increase bone formation [19–22]. PTH (1–34) was also able to regulate osteogenic development via cAMP signaling in a BMP-dependent mesenchymal differentiation system [23]. PTH-induced signaling via G_s_/cAMP pathway plays a particularly important role in skeletal function.

Mesenchymal stem cells are a pluripotent cell type that can differentiate into a variety of cell types, including adipocytes and osteoblasts. Lineage specification is achieved by the expression of transcription factors Cbfa1/Runx2 and Osterix in the case of osteogenesis, and PPARγ in the case of adipogenesis. The number of osteoblasts is partially determined by the lineage specification process. Mice haploinsufficient for PPARγ or Alox15, the lipoxygenase that generates oxidized lipid ligands of PPARγ, have high bone mass phenotype [24, 25]. The control of MSC differentiation into adipocytes and osteoblasts is important in maintaining a normal physiologic bone mass, but the signaling pathways and molecular mechanisms that regulate this process are not well understood.

Cyclic AMP/protein kinase A (PKA) signaling plays a prominent, but ambiguous, role in mesenchymal cell fate decision. Treating human MSCs with PTH inhibited adipocyte development induced by a specific cocktail of adipogenic inducers through the cAMP pathway [26]. Activation of cAMP pathway in calcifying vascular cells derived from primary aortic medial cell cultures resulted in mineralization, enhanced alkaline phosphatase activity, and osteoblast-like differentiation [27]. Pretreatment of human MSCs with a cAMP analog or forskolin, followed by transplantation, enhanced in vivo bone formation [28, 29]. Specific type 4 phosphodiesterase inhibitors could stimulate fibroblast-colony formation, alkaline phosphatase activity, and calcium deposition by rat bone marrow cells [30]. In contrast, a high-throughput siRNA library screen identified components of cAMP pathway as osteogenic suppressors in human MSCs [31]. Activation of PKA by IBMX and forskolin in transformed human MSCs enhanced adipogenesis, while downregulated gene expression of osteogenic gene markers by suppressing leptin [32]. In another one of their reports, de Boer and coworkers [33] observed that cAMP signaling in MSCs was inhibitory on osteogenesis in rodent models, arguing that the difference seen in cAMP-stimulated osteogenic responses could be species-specific.

The role of cAMP pathway in mesenchymal cell fate decision and the subsequent cellular differentiation may depend on the developmental stage in which the cAMP signal is presented. It is important to consider the precise developmental stage of the cells in which the cAMP signal is activated. Activating cAMP signal in differentiating osteoblasts may promote a different osteogenic response from activating cAMP signal in MSCs. Likewise activating cAMP signal in differentiating adipocytes may also promote a different adipogenic response from activating cAMP signal in MSCs. Therefore, we sought to investigate the role of the cAMP signaling in regulating the differentiation of MSCs into mature osteoblasts and mature adipocytes.

## Materials and methods

### Bone marrow stromal cell (BMSCs) cultures

Primary bone marrow stromal cells were isolated from the femurs and tibiae of 8- to 10-week-old FVB/N male mice (The Jackson Laboratory). The cells were collected in primary culture medium (PCM) consisting of α-modification of Eagle’s medium, 10 % fetal bovine serum, 100 U/ml penicillin, 100 μg/ml streptomycin, and 0.25 μg/ml Fungizone (Gibco Cell Culture), and plated onto 10-cm cell culture dishes at a density of 7 × 10^6^ cells/dish. Cells were incubated at 37 °C with 5 % CO_2_ and maintained undisturbed for five days to allow for cell attachment.

### PTH, forskolin, and Wnt3a administrations

Human PTH [1–34] (Bachem product # H-4835), forskolin (Sigma product # F6886), and recombinant mouse Wnt3a (R&D Systems product # 1324-WN) were dissolved in acetic acid (4.07 μM final concentration), ethanol (0.82 % final concentration), and PBS containing 0.1 % bovine serum albumin, respectively.

### Day 5 to day 23 PTH treatment

After incubating BMSCs in PCM for five days, PCM was removed along with all non-adherent cells. The remaining adherent cells containing MSCs were exposed to 10^−7^ M hPTH in osteoblast differentiation medium (see “Initiation of osteoblast differentiation” section) from day 5 to day 23.

### Day 5 to day 10 forskolin treatments

After removal of non-adherent cells, adherent MSCs were exposed to forskolin at final concentrations of 0.001, 0.01, 0.05, and 0.1 mM from day 5 to day 10 in osteoblast differentiation medium.

### Day 0 to day 5 PTH, forskolin, and Wnt3a treatments

**Fig. 1:**
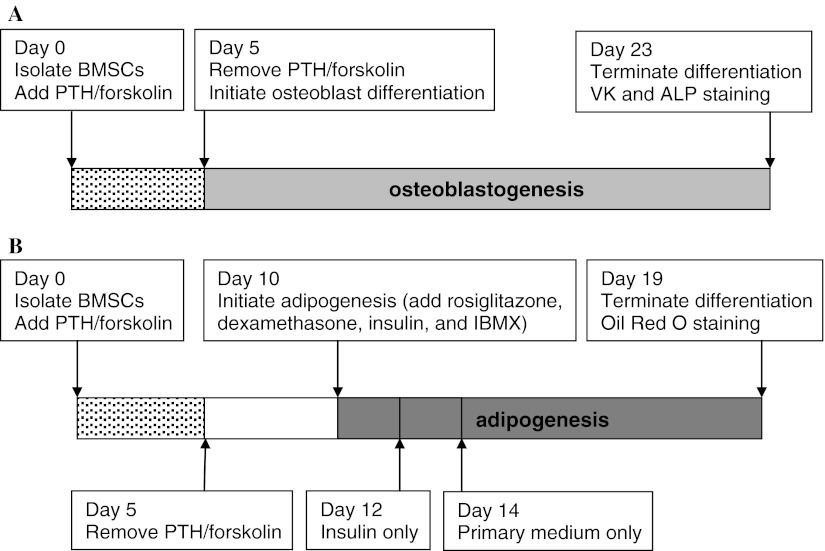
In vitro mesenchymal stem cell differentiation protocols to evaluate the effects of PTH and forskolin stimulation of cAMP signal in MSCs on subsequent osteogenesis or adipogenesis. **a** Mouse BMSCs were freshly isolated from long bones and plated in primary culture medium in the presence of PTH or forskolin and their respective vehicle controls. At day 5, PTH or forskolin was removed and cells were induced to differentiate into osteoblasts. Osteoblastogenesis was allowed to continue until it was terminated on day 23. **b** BMSCs were freshly isolated and plated in primary medium in the presence of PTH or forskolin and their respective vehicle controls. At day 5, PTH or forskolin was removed, and fresh medium was added to the cells. At day 10, adipogenesis was initiated by the addition of rosiglitazone, dexamethasone, insulin, and IBMX. Differentiation medium was replaced on day 12 with medium containing insulin only. Insulin was removed on day 14 and cells were cultured in primary medium until day 19

Freshly isolated BMSCs were exposed to hPTH and forskolin at the above-mentioned concentrations from day 0 to day 5 in PCM (Fig. 1a, b). Freshly isolated BMSCs were exposed to Wnt3a at a final concentration of 25 ng/ml from day 0 to day 5 in PCM (Fig. 1a).

### Day 0 to day 10 forskolin treatments

Freshly isolated BMSCs were exposed to forskolin at final concentrations of 0.001, 0.01, 0.05, and 0.1 mM from day 0 to day 5 in PCM. PCM containing forskolin was removed at day 5 along with non-adherent cells. Adherent cells were exposed to forskolin at these concentrations from day 5 to day 10 in osteoblast differentiation medium.

### Initiation of osteoblast differentiation

After primary BMSCs had been maintained in primary medium for five days, the medium was aspirated and replaced with secondary osteogenic differentiation medium (primary medium containing 50 μg/ml ascorbic acid and 3 mM β-glycerolphosphate) to initiate osteoblast differentiation. Thereafter, secondary differentiation medium was replaced every two or three days (Fig. 1a).

### Initiation of adipogenesis

BMSCs were plated in 6-well plates at 1 × 10^7^ cells/well and remained in PCM for 10 days. At day 10, the medium was replaced with secondary adipogenic differentiation medium (addition of 1 μM rosiglitazone, 1 μM dexamethasone, 5 μg/ml insulin, and 500 μM IBMX), and cells were maintained in it for 2 days. After 2 days, this medium was replaced with PCM containing 5 μg/ml insulin, and cells were incubated in it for another 2 days. Cells were then maintained in PCM until day 19 (Fig. 1b).

### Von Kossa and alkaline phosphatase (ALP) staining of osteoblasts and mineralized nodules

The presence of alkaline phosphatase activity was identified using Leukocyte Alkaline Phosphatase kit (Sigma product # 85L2-1KT). Two percent silver nitrate (Sigma product # S8157) solution was added to cell culture dishes for Von Kossa staining and UV-crosslinked for 10 min. Staining solution was then aspirated, and stained dishes were rinsed twice with distilled water. Stained cultures were scanned and quantified by means of Improvision Openlab software version 5.0.2.

### Oil Red O staining for lipid accumulation in differentiated cells

Cells in 6-well plates were fixed in PBS containing 10 % formaldehyde. Following fixation, cells were stained in Oil Red O for 15 min. Cells were rinsed several times with distilled water to remove excess stain. Stained cultures were scanned and quantified by means of Improvision Openlab software version 5.0.2.

### RNA extraction and quantitative real-time PCR (qRT-PCR)

Total RNA from BMSCs and osteoblast cultures were isolated using PureLink Micro-to-Midi total RNA purification system (Ambion Cat. # 12183-018) and further purified using RNeasy Mini Kit (Qiagen Cat. # 74104). Reverse transcription was carried out using TaqMan Reverse Transcription Reagents (Applied Biosystems Part # N808-0234). Gene expression was quantified by SYBR Green I-based quantitative real-time PCR utilizing SYBR Green PCR Master Mix (Applied Biosystems part # 4309155). Primers were synthesized by Elim Biopharmaceuticals, Inc. (Table 1). PCR was carried out in Applied Biosystems 7900HT real-time thermocycler. The results were analyzed by means of 7300 System SDS version 1.4 supplied with the machine.

**Table 1:** Sequence of primers used in qRT-PCR

Gene	Forward primer	Reverse primer
Runx2	CGAGACCAACCGAGTCATTT	ACGCCATAGTCCCTCCTTTT
Osterix	TTTCTCATTAACTCGTTGCCATCT	CTTCGGGAAAACGGCAAATA
Collagen 1a1	GCGAAGGCAACAGTCGCT	CTTGGTGGTTTTGTATTCGATGAC
Osteocalcin	CTGACCTCACAGATGCCAAG	GTAGCGCCGGAGTCTGTTC
Alkaline phosphatase	GCACCTGCCTTACCAACTCT	TGGAGTTTCAGGGCATTTTT
Axin 2	TGACTCTCCTTCCAGATCCCA	TGCCCACACTAGGCTGACA
Lef 1	AGAAATGAGAGCGAATGTCGTAG	CTTTGCACGTTGGGAAGGA
Tcf 1	GTTCACCCACCCATCCTTGAT	TGCCTGTGTTTTCAGGTTTCT

### Statistical analysis

Data are presented as mean ± standard error of at least three independent experiments. Statistical differences between the means were examined by Student’s *t* test by means of GraphPad Prism 5 software, and significance was set at a *P* value of <0.05.

## Results

### Effects of PTH and forskolin activation of cAMP signaling in differentiating osteoblasts on osteogenesis

**Fig. 2:**
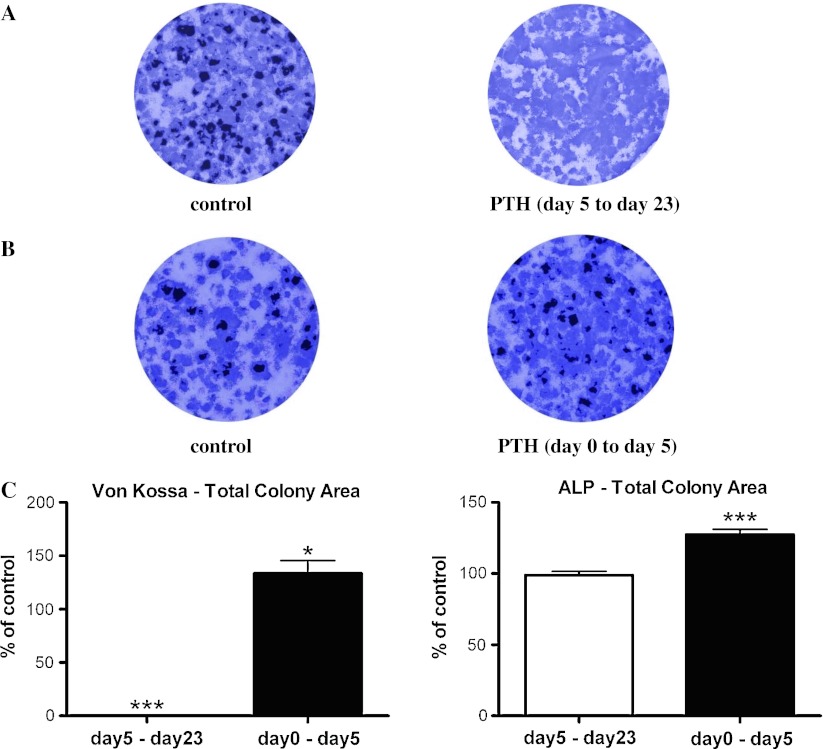
Long-term exposure to PTH during osteogenesis completely abolished mineralization, but exposure to PTH for five days before initiation of osteogenesis stimulated osteogenesis. **a** Von Kossa staining showed no mineralized nodules in cultures that were continuously exposed to 10^−7^ M hPTH [1–34] from day 5 to day 23. (Von Kossa-positive mineralized nodules are stained *black*. Alkaline phosphatase (ALP)-positive areas are stained *purple*.) **b** Exposure to PTH from day 0 to day 5 modestly increased both total Von Kossa-stained area and total ALP-positive area. **c** Total colony area positive for Von Kossa staining and total ALP-positive colony area were quantified as percent of basal level of control. Data are mean ± SEM. *P < 0.05 and ***P < 0.001 vs. control; *n* = 3. *Unfilled bars* represent osteoblast cultures that were exposed to PTH from day 5 to day 23. *Filled bars* represent BMSC cultures that were exposed to PTH from day 0 to day 5 prior to induction of osteogenesis

To test the effects of long-term exposure to PTH on differentiating osteoblasts, we exposed osteoblasts continuously to PTH from the time when osteoblast differentiation (day 5) was induced until it was terminated (day 23). Continuous long-term exposure to PTH completely abolished the appearance of Von Kossa-stained mineralized nodules in the cell culture (Fig. 2a). However, total ALP activities in culture was not affected by exposure to PTH (Fig. 2a, c), suggesting that continuous PTH treatment on differentiating osteoblasts primarily affected osteoblasts’ ability to mineralize.

**Fig. 3:**
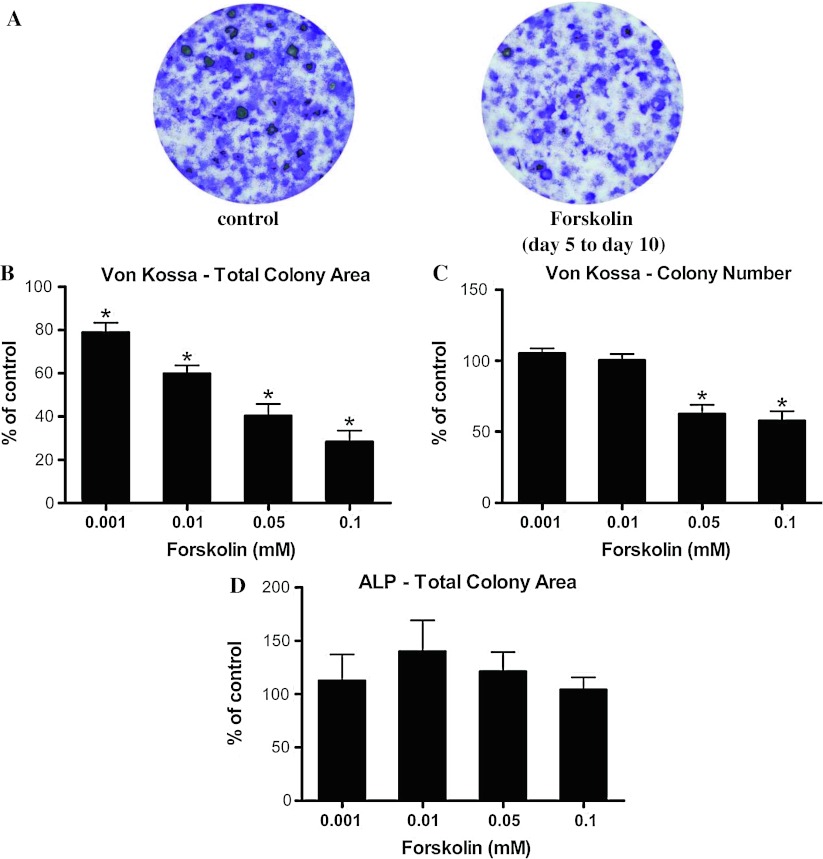
Five-day treatment with forskolin after initiation of osteoblast differentiation diminished osteoblast mineralization. **a** Exposure to 0.1 mM forskolin immediately after initiation of osteogenesis for 5 days reduced Von Kossa staining of mineralized nodules, **b** total Von Kossa-positive colony area, and **c** total Von Kossa-positive colony number. **d** No changes in ALP-positive total colony area were detected. *Data* were quantified as percent of basal level of control and represent mean ± SEM. *P < 0.05 vs. vehicle-treated controls; *n* = 3

To determine whether the effects of PTH on differentiating osteoblasts were exerted through the cAMP/PKA pathway, we pharmacologically stimulated cAMP signaling using an activator of adenylate cyclase, forskolin. Exposing osteoblasts to different concentrations of forskolin immediately after initiation of osteoblast differentiation for five days (from day 5 to day 10) significantly reduced the total mineralized area and total mineralized colony number (Fig. 3a). This decrease in total mineralized area and total mineralized colony number was dosage dependent (Fig. 3b, c). However, total ALP activity in culture was not affected (Fig. 3d), similar to what was observed in osteoblast cultures that were treated with PTH continuously from day 5 to day 23. We also examined the effects of long-term intermittent exposure to forskolin (cells in the presence of forskolin every other two days or 2 h per day) from day 5 to day 23. Although Von Kossa staining was completely abolished, ALP activity could not be accurately measured as the cells appeared to be dying from the toxic effects of long-term exposure to forskolin (data not shown). Therefore, we concluded that a transient elevation in cAMP signaling in differentiating osteoblasts negatively regulated mineralization, but had no effect on the number of ALP-positive cells.

### Effects of PTH and forskolin activation of cAMP signaling in BMSCs on subsequent osteogenesis

To examine the effects of exposing MSCs to PTH on the subsequent osteoblast differentiation, freshly isolated BMSCs were exposed to PTH for five days. Before the induction of osteoblast differentiation, cells from bone marrows that had attached to culture dishes were allowed to differentiate later in the absence of PTH (Fig. 1a). We presumed that most of the attached cells were of mesenchymal origin and likely MSCs. BMSCs that were exposed to PTH were able to produce a 34 % increase in mineralization and a 27 % increase in the total level of ALP activity over vehicle-treated control cultures (Fig. 2b, c). Thus, treating BMSCs with PTH enhanced the ability of osteoblasts to mineralize and increased the number of ALP-positive cells.

**Fig. 4:**
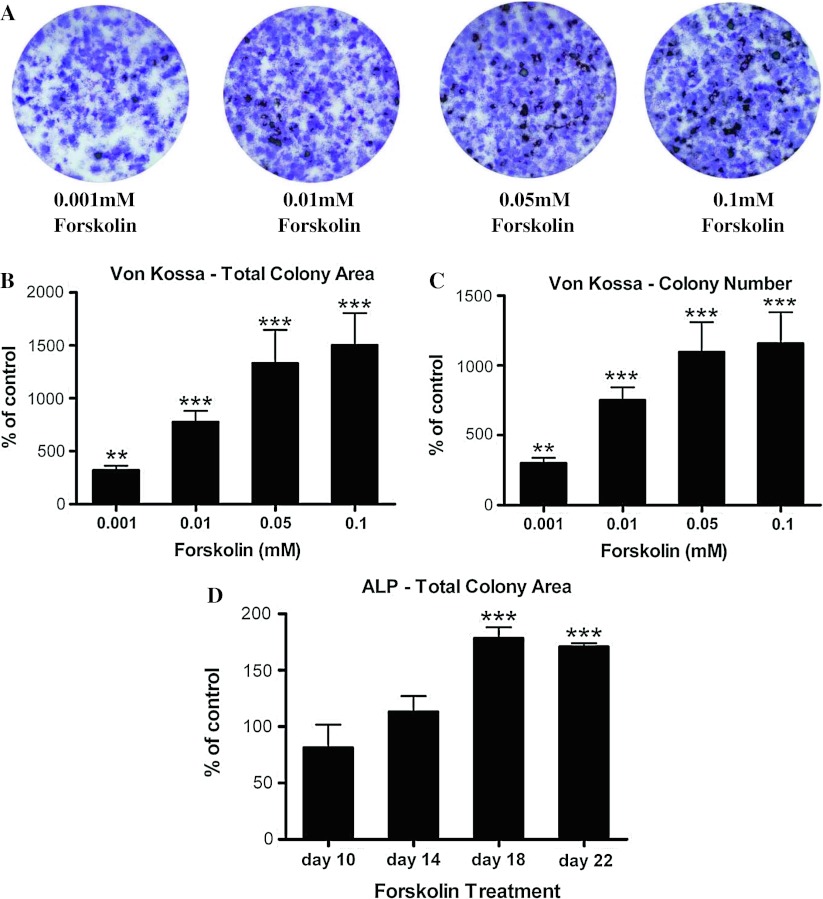
Treatment of MSCs with forskolin followed by induction of osteoblast differentiation resulted in a dose-dependent increase in osteogenesis. Primary mouse BMSCs were exposed to different concentrations of forskolin for five days before induction of osteoblast differentiation. **a** Exposure to forskolin during the 5-day period was able to promote more efficient osteogenesis as indicated by increased Von Kossa staining of mineralized nodules at day 23. **b** Total colony area stained positive for Von Kossa staining and **c** total Von Kossa-positive colony number were quantified as percent of basal level of control. **d** Osteoblast differentiation was terminated at day 10, day 14, day 18, and day 22 to detect for ALP activity at different time points of osteoblast differentiation. Treatment of BMSCs with forskolin followed by induction of osteoblast differentiation resulted in an increase in ALP-positive colony area by day 18. ALP activity was quantified as percent of basal level of control. Data are mean ± SEM. **P < 0.01 and ***P < 0.001 vs. vehicle-treated controls; *n* = 3

We then investigated the effects of forskolin treatment of BMSCs on subsequent osteoblast differentiation. Freshly isolated BMSCs were immediately exposed to different doses of forskolin for five days before the initiation of osteogenesis (Fig. 1a). Exposure to forskolin during this five-day period promoted an increased osteogenesis as measured by Von Kossa staining of mineralized nodules (Fig. 4a). This increase in the mineralization was dose dependent (Fig. 4b, c). Forskolin treatment resulted in a 78 % increase in total ALP activity over vehicle-treated control by day 18 (Fig. 4d). Similar to PTH, exposing BMSCs to forskolin increased mineralization as well as ALP-positive cell numbers.

**Fig. 5:**
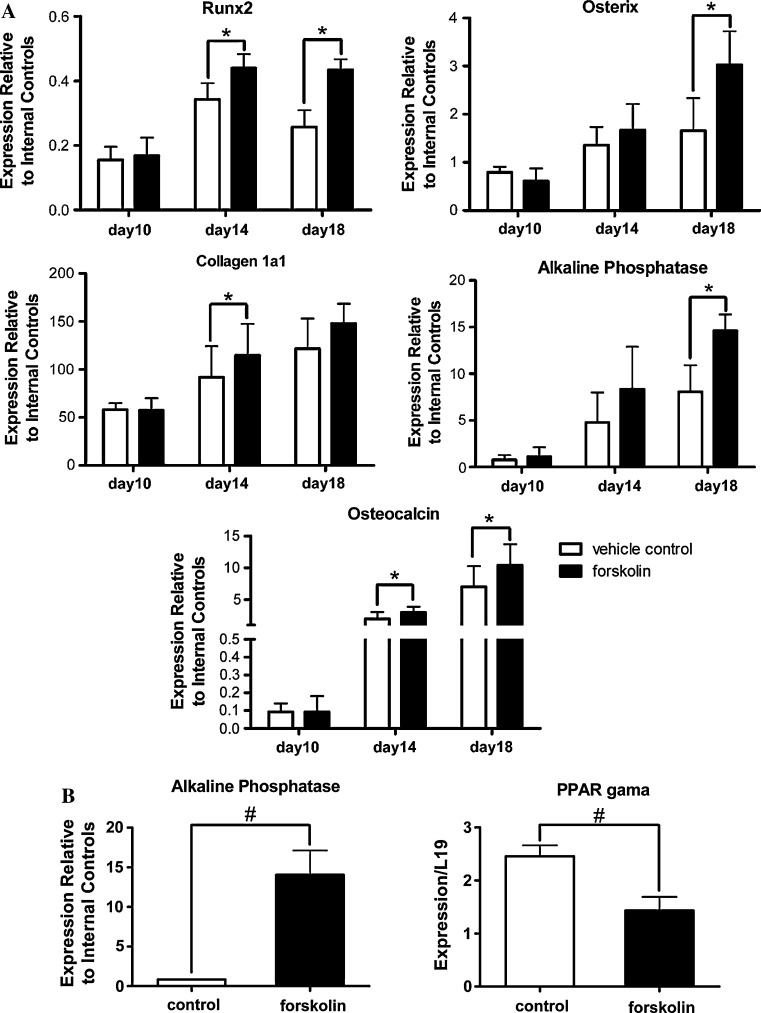
**a** Treatment of MSCs with forskolin followed by induction of osteogenesis resulted in upregulated expression of osteoblast marker gene in late stages of osteoblast differentiation. MSCs were exposed to 0.1 mM of forskolin from day 0 to day 5 before induction of osteoblast differentiation. RNA was isolated from day 10, 14, and 18 of osteoblast cultures. Quantitative real-time RT-PCR was carried out to detect changes in marker gene expression as a result of forskolin treatment. Transcript levels were quantified relative to the average of the expression levels of the internal housekeeping genes, L19 and Hprt1. Data are mean ± SEM. *P < 0.05; *n* = 3. *Unfilled bars* represent control MSC cultures that were not exposed to forskolin while *filled bars* represent cultures that were exposed to forskolin from day 0 to day 5 prior to induction of osteogenesis. **b** ALP gene expression was upregulated, while PPARγ gene expression was downregulated in MSCs following five days of exposure to forskolin. Reverse transcription and real-time qPCR were performed on RNA isolated from MSCs treated with 0.1 mM forskolin or 10^−7^ M hPTH [1–34] and their respective vehicle controls from day 0 to day 5. ALP transcript levels were quantified relative to the average of the expression levels of the internal housekeeping genes, L19 and Hprt1. PPARγ transcript levels were quantified relative to the expression of L19. Data are mean ± SEM. ^#^ *P* < 0.05; *n* ≥ 3

Activation of cAMP signaling in MSCs by forskolin for five days promoted a greatly enhanced osteogenesis. Concurrently, there was an increase in the expression of genes associated with osteoblast differentiation. Quantitative real-time PCR demonstrated a significant increase in the level of transcripts encoding Runx2 and osteocalcin at day 14 and day 18 over the vehicle-treated controls as a result of forskolin treatment (Fig. 5a). Transcripts encoding Osterix and ALP were also significantly increased at day 18, as compared to the controls (Fig. 5a). Collagen 1α1 gene expression was significantly elevated at day 14 but not at day 18 (Fig. 5a).

### Combined effects of forskolin activation of cAMP signaling in BMSCs and differentiating osteoblasts on osteogenesis

**Fig. 6:**
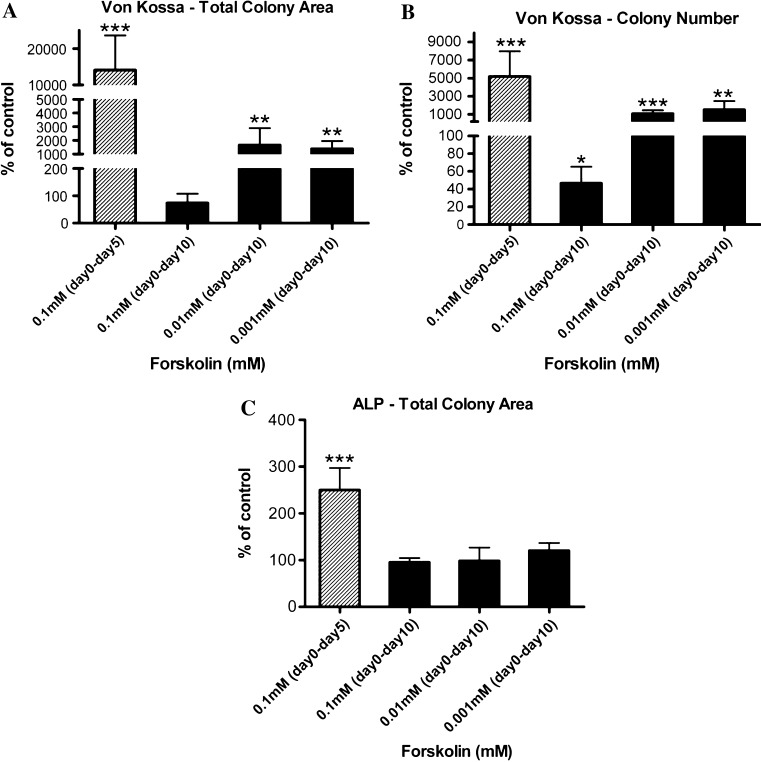
Five-day forskolin-activated cAMP signaling in differentiating osteoblasts could suppress increased mineralization resulting from forskolin-activated cAMP signaling in BMSCs. Primary mouse BMSCs were exposed to different concentrations of forskolin for five days before induction of osteoblast differentiation followed by continued forskolin treatment after induction for further five days. **a** Continued 0.1 mM forskolin treatment after induction of differentiation reduced total Von Kossa-positive colony area, **b** Von Kossa-positive colony number but not **c** total ALP-positive colony area. *Data* were quantified as percent of basal level of control and represent mean ± SEM. *P < 0.05, **P < 0.01, and ***P < 0.001 vs. vehicle-treated controls; *n* = 3

To determine whether the anabolic effect of activated cAMP signaling in BMSCs on subsequent osteogenesis could be maintained in differentiating osteoblasts receiving continuously activated cAMP signaling, BMSCs were treated with forskolin from day 0 to day 5, and remaining adherent cells were continuously exposed to freshly added forskolin from day 5 to day 10, after induction of osteogenesis. Continued exposure to forskolin at 0.1 mM for five days after initiation of osteogenesis completely suppressed the increase in total Von Kossa-positive colony area (Fig. 6a) stimulated by five-day 0.1 mM forskolin exposure before osteogenic induction. Continued exposure to forskolin at this dose also significantly reduced Von Kossa-positive colony number relative to the vehicle control (Fig. 6b). When cells were exposed to forskolin at lower doses from day 0 to day 10, the anabolic effect of forskolin was maintained as assessed by both Von Kossa-positive colony area and number (Fig. 6a, b). Interestingly, the total ALP-positive colony area resulting from the forskolin treatment at all concentrations was not altered relative to the vehicle control (Fig. 6c). The increase in ALP-positive colony area resulted from 5-day 0.1 mM forskolin activation in BMSCs was suppressed, but not below the level of the vehicle-treated control.

### Effects of PTH and forskolin activation of cAMP signaling in BMSCs on subsequent adipogenesis

**Fig. 7:**
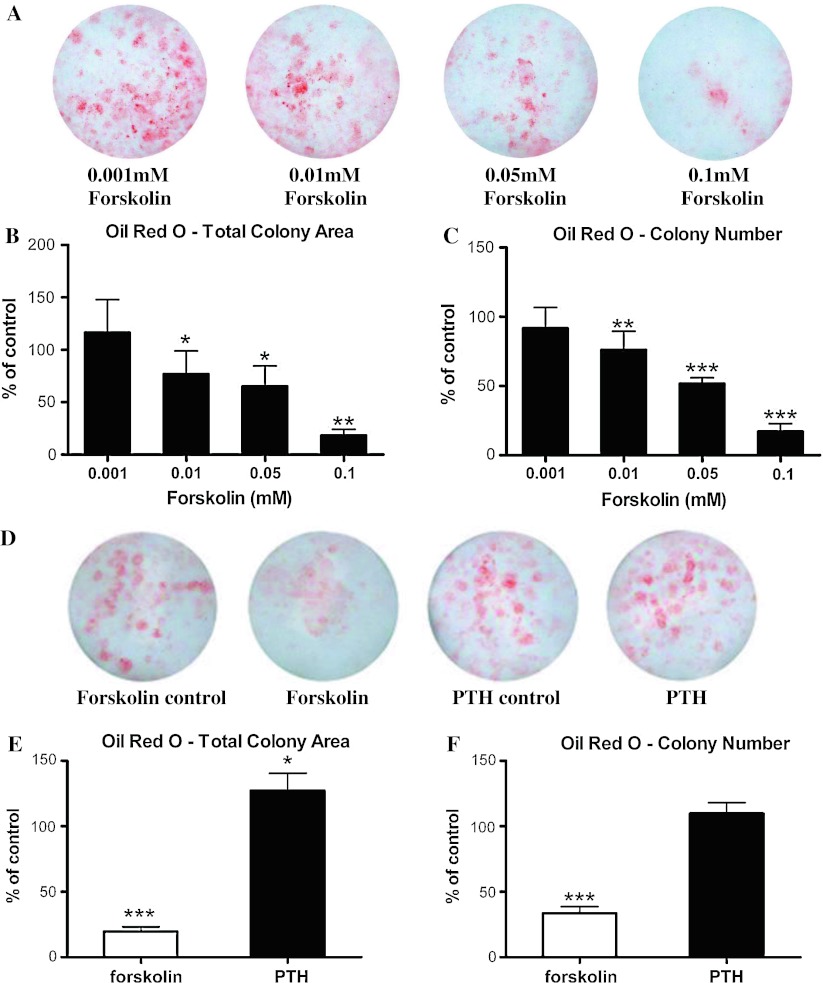
Treatment of MSCs with forskolin followed by induction of adipogenesis resulted in a dose-dependent decrease in adipogenesis, while treatment of MSCs with PTH followed by induction of adipogenesis resulted in a modest increase in adipogenesis. Mouse BMSCs were exposed to different concentrations of forskolin from day 0 to day 5 before induction of adipogenesis at day 10. **a** Oil Red O staining of lipid contents of the cell indicated the degree of adipogenesis. **b** Total colony area stained positive for Oil Red O staining and **c** total Oil Red O-positive colony number were quantified as percent of basal level of control. *Data* are mean ± SEM. *P < 0.05, **P < 0.01, and ***P < 0.001; *n* = 3. Mouse BMSCs were exposed to 0.1 mM of forskolin or 10^−7^ M of hPTH [1–34] from day 0 to day 5 before induction of adipogenesis at day 10. **d** Oil Red O stained for lipid content of the cell. **e** Total colony area stained positive for Oil Red O staining and **f** total colony number stained positive for Oil Red O were quantified as percent of basal level of control. Data are mean ± SEM. *P < 0.05, ***P < 0.001; *n* = 4

Our results suggest that induction of cAMP signaling in pluripotent MSCs favors differentiation to osteogenic cells. If so, then differentiation of other lineages such as adipogenesis might be inversely affected. To test this, we proceeded with the same forskolin treatment, but induced MSCs to become adipocytes. Following our standard protocol, adipogenesis was initiated at day 10, instead of day 5 in the osteogenesis protocol. In contrast to an enhanced osteogenic response, activation of cAMP signaling in MSCs diminished the efficiency of adipogenesis in a dose-dependent manner, as assessed by Oil Red O staining (Fig. 7a, b). There was a dose-dependent decrease in Oil Red O-positive colony number (Fig. 7c), suggesting that activation of cAMP signaling in MSCs promoted more MSCs to commit toward the osteogenic pathway and concomitantly reduced the number of MSCs committed toward the adipogenic pathway.

Next, we examined the effect of exposing BMSCs to PTH on subsequent adipogenesis. PTH treatment on BMSCs for five days actually produced a modest increase (27 %) in the subsequent adipogenesis as measured by Oil Red O staining (Fig. 7d, e). However, the total Oil Red O-positive colony number was not significantly affected (Fig. 7f). Unlike the effects of forskolin, PTH treatment on BMSCs did not reduce the subsequent adipogenesis.

### Expression of early gene markers of osteogenesis and adipogenesis in MSCs following five-day forskolin treatment

Although forskolin treatment of BMSCs increased expression of genes associated with osteoblast differentiation, changes in the expression of these genes might only reflect the altered efficiency of osteoblast differentiation. Critical changes at the molecular level might have already occurred at earlier stages of differentiation process. Therefore, we examined changes in the expression of early marker genes before the induction of differentiation. Transcript levels of three early markers of osteogenesis (Runx2, Osterix, and ALP) and three early markers of adipogenesis (C/EBP, PPARγ, and ADRP) were measured after five-day forskolin or PTH treatment. Exposure to forskolin increased the ALP transcript level in MSCs by 17 fold over vehicle-treated controls (Fig. 5b). On the other hand, PPARγ was downregulated by more than forty percent (Fig. 5b). No significant changes in transcript levels were detected in other genes (data not shown). Five-day exposure to PTH had little impact on the expression of any of these early marker genes in MSCs (data not shown). Thus, forskolin was able to alter the gene expression of ALP and PPARγ prior to the start of differentiation process.

### Canonical Wnt pathway as a mediator of PTH/cAMP action in MSCs

**Fig. 8:**
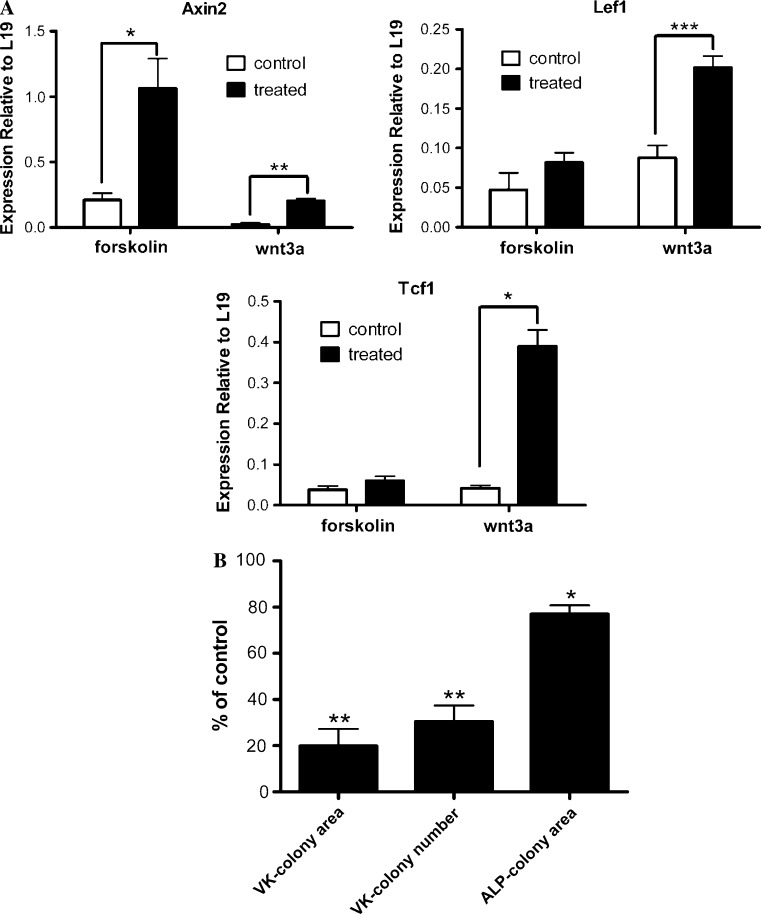
**a** Effects of five-day exposure to forskolin or Wnt3a on Wnt target gene expression in mesenchymal stem cells. MSCs were treated with 0.1 mM of forskolin or 25 ng/ml of Wnt3a and their respective vehicle controls from day 0 to day 5. RNA was isolated on day 5 and subjected to quantitative real-time RT-PCR. Gene expression was quantified relative to the expression of L19. **b** Treatment of MSCs with Wnt3a followed by induction of osteoblast differentiation resulted in decreased osteogenesis. Primary mouse BMSCs were exposed to 25 ng/ml of Wnt3a for 5 days before induction of osteoblast differentiation. Exposure to Wnt3a during the 5-day period produced less efficient osteogenesis as indicated by reduced total Von Kossa-positive colony area, Von Kossa-positive colony number, and ALP-positive colony area. Von Kossa and ALP staining were quantified as percent of basal level of control. Data are mean ± SEM. *P < 0.05, **P < 0.01, and ***P < 0.001 vs. vehicle-treated controls; *n* = 3

Wnt/β-catenin pathway plays an important role in normal bone biology, and deregulation of this process contributes to bone diseases [34–36]. Emerging data suggest that canonical Wnt signaling promotes maturation of osteoblastic precursor cells into mature osteoblasts [37, 38]. To determine whether cAMP signaling exerts its action through Wnt/β-catenin pathway in MSCs, we measured transcript levels of canonical Wnt target genes in MSCs that had been treated with forskolin from the time of plating until day 5. Gene expression of Tcf1 and Lef1 was not significantly altered by forskolin treatment; however, expression of Axin2 was upregulated by five-fold (Fig. 8a). Activating Wnt/β-catenin pathway by exposing MSCs to Wnt3a for the same duration significantly increased expression of Axin2, Tcf1, and Lef1 (Fig. 8a). Expression of these Wnt target genes were also examined in MSCs exposed to PTH. No changes in the transcript levels of these genes were detected (data not shown). Despite an increased expression of Axin2, forskolin activation of cAMP signaling in MSCs did not increase the expression of other classical canonical Wnt target genes, which were upregulated by Wnt3a. When mineralization of osteoblasts was examined following 5-day Wnt3a treatment on BMSCs, Wnt3a actually conditioned the cells to less efficiently differentiate into mature osteoblasts, as indicated by reduced total Von Kossa-positive colony area, Von Kossa-positive colony number, and ALP-positive area (Fig. 8b). The effects of exposing BMSCs to forskolin or PTH on subsequent osteogenesis were not mediated by the canonical Wnt signaling pathway.

## Discussion

Precisely, how G_s_-mediated cAMP signaling regulates MSC development remains controversial. In this paper, we explored the direct involvement of cAMP pathway in regulating MSC differentiation and demonstrated that stimulating cAMP signaling in MSCs enhanced osteogenic response, while continuous activation of cAMP signaling in differentiating osteoblasts negatively affected mineralization. Furthermore, stimulating cAMP signaling in MSCs favored osteogenic development over adipogenic development.

First, we used natural ligand, PTH, to activate the cAMP pathway in developing osteoblasts and MSCs. Using primary mouse BMSCs as the source of MSCs, we tested the effects of long-term exposure to PTH after initiation of osteogenesis. Long-term exposure to PTH during differentiation completely abolished the osteoblasts’ ability to mineralize, but not the number of ALP-positive colonies. On the other hand, treatment of BMSCs with PTH, followed by induction of osteogenesis, resulted in a modest increase in osteogenesis. As such, PTH produced varied effects on the mineralization potential of osteoblasts depending on the developmental stage of the cells.

PTH can also activate the MAPK cascade through G_i_, as well as the phospholipase C pathway via G_q_ signaling. Therefore, forskolin was used to directly stimulate the production of cAMP to examine the specific role of cAMP signaling pathway in MSC differentiation. Stimulation of cAMP signaling by forskolin in differentiating osteoblasts for five days resulted in diminished osteogenesis, both in terms of total area of mineralization and number of mineralizing colonies. Thus, exposing differentiating osteoblasts to PTH or forskolin greatly reduced osteoblasts’ ability to mineralize.

Direct activation of cAMP signaling by forskolin in BMSCs yielded an osteogenic response that was opposite of activating cAMP signaling in differentiating osteoblasts. In addition to increased mineralization, there was an increase in the number of ALP-positive colonies. Dosage-dependent increase in the ratio of Von Kossa signal over ALP signal (data not shown) indicated that cAMP signaling in BMSCs conditioned the cells to more efficiently differentiate into osteoblasts, particularly their ability to mineralize. Elevated expression of osteoblast marker genes such as Runx2, Osterix, Collagen 1α1, Osteocalcin, and ALP at the late stages of differentiation also implied a more efficient osteoblast differentiation. As to why modest increase in gene expression of Collagen 1α1 and Osteocalcin could result in strong mineralization, one possibility is that early expression of ALP (before osteogenic induction) could act to increase local concentration of inorganic phosphate, a mineralization promoter, and to decrease the concentration of extracellular pyrophosphate, an inhibitor of mineral formation [39]. Conversely, the effect of direct activation of cAMP signaling in BMSCs on adipogenesis was negative. Activation of cAMP signaling in mesenchymal cells, which were earlier in their development, might impart in them an ability to more efficiently differentiate into mature osteoblasts and less efficiently differentiate into mature adipocytes.

Activation of cAMP signaling by forskolin in BMSCs produced increased number of ALP-positive osteogenic colonies and decreased number of adipogenic colonies as measured by total Oil Red O staining. Expressions of early osteogenic and adipogenic marker genes were examined in MSCs which had been exposed to forskolin to determine changes in osteogenic or adipogenic potential of MSCs. ALP gene expression was greatly upregulated in MSCs that had been exposed to forskolin although the same treatment with PTH did not alter its expression. Conversely, PPARγ gene expression was downregulated in MSCs by forskolin treatment. Interestingly, the elevated ALP transcript level was not maintained once cells had been induced to differentiate into osteoblasts (Fig. 5, day 10) and recovered only in later stages of osteogenesis (Fig. 5, day 18). We do not know the biologic relevance of this transcriptional response. Perhaps, ALP transcriptional activation requires continuous presence of forskolin, thus it could be a direct target of cAMP signaling. Sustained elevated cAMP signaling in MSCs might have produced epigenetic changes that altered the transcriptional control of specific promoters. Recently, a PKA-dependent histone lysine demethylase complex had been characterized, suggesting that PKA signal could regulate histone methylation and gene transcription [40]. This could be a potential mechanism that cAMP acts through to favor osteogenesis over adipogenesis in MSCs. Forskolin did increase PCNA transcript levels in MSCs (data not shown), suggesting changes in the makeup of MSC population. As such, activation of cAMP signaling in BMSCs could be both pro-osteogenic and mitogenic, resulting in more MSCs directed toward osteogenic lineage and less MSCs directed toward adipogenic lineage.

PTH can control Wnt signaling in osteoblast lineage cells via regulation of negative modulators such as Dkk1 and Sclerostin [41, 42]. There is also evidence that the effect of PTH on the canonical Wnt pathway occurs at least partially through the cAMP/PKA signaling by differentially regulating Wnt receptor complex proteins and Dkk1 [43]. The role of canonical Wnt signaling in mesenchymal cell’s ability to differentiate into osteoblasts is unclear. Wnt stimulation had been reported to regulate osteogenesis in human MSCs both negatively and positively in a context-specific manner [44]. Maturation state of the osteogenic cells also appeared to be important. Activation of canonical Wnt signal in undifferentiated MSCs and juvenile mouse calvarial osteoblasts inhibited osteogenesis, but strongly induced osteogenesis in mature calvarial osteoblasts [45, 46]. When we measured canonical Wnt target gene expression in MSCs exposed to either forskolin or PTH for 5 days before induction of osteoblast differentiation, only Axin2 gene expression was significantly upregulated by forskolin treatment. We also treated MSCs with Wnt3a for five days to make sure the gene expression of the selected Wnt target genes could be controlled by Wnt3a. As expected, Wnt3a was able to upregulate selected Wnt target genes. Contrary to enhanced osteogenic response produced by forskolin, osteogenic response followed by 5-day Wnt3a treatment was greatly reduced. Therefore, the effect of activation of cAMP signaling in MSCs on subsequent osteogenesis was not mediated through the canonical Wnt pathway.

Previously, several groups had investigated the role of cAMP in MSC linage specification. A high-throughput siRNA library screen conducted by Zhao and Ding [31] identified *GNAS* (G_αs_) and *ADCY8* (adenylate cyclase 8) as genes that suppressed osteogenic specification. The authors carried out a very similar experiment in which cells were subjected to specific siRNA knockdowns (initial 2–4 days) followed by osteogenic induction (additional 5–9 days) [31]. Gene expression knockdown of these targets genes enhanced osteogenic maturation. Substituting siRNA with forskolin suppressed osteogenesis although a human MSC cell line (PT-2501) was used in their experiments [31]. In contrast, de Boer and coworkers [28, 29] pretreated human MSCs derived from bone marrow with db-cAMP or forskolin in basic medium and was able to enhance in vivo bone formation after transplantation. Subsequently, they proceeded to test the same idea in the rodent models. Db-cAMP was applied to mouse MSCs in the presence of mineralization medium for 3 or 10 days. These treatments suppressed mineralization [33]. As noted, db-cAMP was present in the mineralization medium in one case but not the other. It is important to consider the specific type of cell population in which the cAMP signal is activated, whether it is homogeneous MSCs, MSCs populated with other elements of the bone marrow, or MSCs that have been induced to undergo osteogenesis. In addition, dexamethasone was used in these experiments as one of the osteogenic inducers, while it was not used in our study. It is a synthetic member of the glucocorticoid class of steroid hormones and can exert its influence on lineage commitment through a cAMP/PKA-independent mechanism [47].

PTH and forskolin treatments on BMSCs sometimes produced different effects on subsequent MSC development. Stimulating cAMP signal in BMSCs by forskolin reduced the efficiency of adipogenesis, whereas PTH enhanced adipogenesis to a small degree. Forskolin activation of cAMP signaling in BMSCs favored osteogenesis over adipogenesis. Exposure to PTH promoted both. This is in contrast to the findings by Rickard et al. [26] where intermittent PTH treatment or a non-peptide agonist of PTH1 receptor suppressed adipocyte differentiation from human MSCs. However, human MSCs used in these experiments were relatively homogeneous. In contrast, BMSCs derived from mouse long bones are heterogeneous, and it is possible that cells other than MSCs contribute to the ability of PTH to promote adipogenesis. Furthermore, Rickard’s human MSCs were cultured and treated with PTH under mildly adipogenic conditions whereas we activated PTH and cAMP signaling before adipogenic induction. Finally, in our case, PTH and forskolin treatments were continuous for five days. In Richard’s experiments, intermittent PTH and forskolin treatments were one hour per day. Further studies are needed to define the precise basis for the differences between the present results and those of Rickard’s et al. [26].

PTH stimulates a diverse range of signaling pathways. It would be interesting to pharmacologically inhibit either cAMP/PKA or protein kinase C/MAPK pathway to discover the contribution of each signaling pathway to the effects of PTH on osteogenesis and adipogenesis. However, the different results produced by PTH and forskolin can also be attributed to the cell types which express PTH1R to receive the signal. Forskolin treatment of BMSCs activated cAMP pathway in all cell types in that population. PTH treatment activated cAMP pathway only in cells that express PTH1R. Therefore, without knowing the cell populations that express PTH receptors, it is difficult to interpret results of pharmacological manipulation of the downstream pathways of PTH signaling. BMSCs consist of a wide range of cell types, including cells of hematopoietic lineage. Studies using genetically modified mice provided evidence that PTH-mediated anabolic response required participation of cells of hematopoietic origin [48–50]. Osteopetrotic c-fos-deficient mice that have osteoclast defect were not responsive to PTH treatments [49]. Perhaps it is more important to first identify the cell type(s) in which activated cAMP signaling can potentiate osteogenesis and to identify the GPCR(s) that activates cAMP signaling and can induce a more robust osteogenesis. Recent reports suggested that G_s_- and G_q_-coupled A2A and A2B adenosine receptors played a role in promoting proliferation and differentiation of bone marrow-derived mesenchymal stem cell [51, 52]. In fact, A2B adenosine receptor knockout mice exhibited lower bone density in the femur [52]. Moreover, transfection of A2B receptors into murine osteoblast precursor cell line, 7F2, stimulated osteocalcin and ALP expression and inhibited subsequent adipogenesis [53].

In conclusion, activation of cAMP pathway in BMSCs conditioned MSCs to produce a more efficient osteogenic response, but a less efficient adipogenic response. On the other hand, sustained activation of cAMP pathway in differentiating osteoblasts inhibited mineralization. Cyclic AMP signaling in BMSCs may have a role in controlling the balance between adipogenesis and osteogenesis, partially by regulating lineage commitment of MSCs.
